# Dynamic and Flexible Survival Models for Extrapolation of Relative Survival: A Case Study and Simulation Study

**DOI:** 10.1177/0272989X221107649

**Published:** 2022-06-29

**Authors:** Benjamin Kearns, Matt D. Stevenson, Kostas Triantafyllopoulos, Andrea Manca

**Affiliations:** The University of Sheffield, Sheffield, UK; The University of Sheffield, Sheffield, UK; The University of Sheffield, Sheffield, UK; The University of York, York, UK

**Keywords:** economic evaluation, forecasting, parametric survival models, relative survival

## Abstract

**Background:**

Extrapolation of survival data is a key task in health technology assessments (HTAs), which may be improved by incorporating general population mortality data via relative survival models. Dynamic survival models are a promising method for extrapolation that may be expanded to dynamic relative survival models (DRSMs), a novel development presented here. There are currently neither examples of dynamic models in HTA nor comparisons of DRSMs with other relative survival models when used for survival extrapolation.

**Methods:**

An existing appraisal, for which there had been disagreement over the approach to survival extrapolation, was chosen and the health economic model recreated. The sensitivity of estimates of cost-effectiveness to different model choices (standard survival models, DSMs, and DRSMs) and specifications was examined. The appraisal informed a simulation study to evaluate DRSMs with relative survival models based on both standard and spline-based (flexible) models.

**Results:**

Dynamic models provided insight into the behavior of the trend in the hazard function and how it may vary during the extrapolated phase. DRSMs led to extrapolations with improved plausibility for which model choice may be based on clinical input. In the simulation study, the flexible and dynamic relative survival models performed similarly and provided highly variable extrapolations.

**Limitations:**

Further experience with these models is required to identify settings when they are most useful, and they provide sufficiently accurate extrapolations.

**Conclusions:**

Dynamic models provide a flexible and attractive method for extrapolating survival data and facilitate the use of clinical input for model choice. Flexible and dynamic relative survival models make few structural assumptions and can improve extrapolation plausibility, but further research is required into methods for reducing the variability in extrapolations.

Health technology assessment (HTA) is the scientific evaluation of health technologies and informs decisions regarding whether a health technology should be funded. For consistent decision making, all relevant costs and consequences associated with the appraised technology should be included in the HTA. When the treatment affects survival, it is important that lifetime outcomes be included in the assessment.^1^ Estimates of lifetime mean survival typically require extrapolations of incomplete survival functions. These estimates can be key drivers of estimates of cost-effectiveness and hence funding decisions.^2^ This illustrates the importance of using appropriate methods for extrapolation.

A recent review of methods for extrapolating survival data in cancer appraisals concluded that current approaches were “suboptimal,” with an overreliance on common survival models, which may not adequately capture the complexities of hazard functions that are expected to arise from clinical trials.^3^ Dynamic survival models (DSMs) have recently been suggested as flexible models for the analysis and extrapolation of survival data.^4^ These may be viewed as relaxing the structural assumptions of common survival models by allowing their parameters to vary over time, with this temporal variation modeled by a time series. A particular advantage of DSMs is that extrapolations are based on all the data while simultaneously giving more weight to more recent observations. This resolves the disagreement in the literature over how much evidence should be included when generating extrapolations.^5–8^ Despite these advantages of DSMs, there is a dearth of examples of their use in HTA.

Another approach to improve extrapolations is via the incorporation of external long-term evidence, such as general population mortality data.^9–13^ In particular, additive relative survival models decompose the overall hazard function into the sum of disease-specific (or “excess”) hazards and general population hazards. Extrapolations are obtained for the former, and the additive structure ensures that the overall hazard function never falls below the general population hazards. Models for the disease-specific hazard function include standard parametric models and flexible spline-based models.^14–17^ In addition, DSMs may be used, providing dynamic relative survival models (DRSMs), a novel method that has not previously been evaluated.

This article has 2 primary objectives. The first is to demonstrate the use of DSMs and DRSMs in HTA via a reanalysis of an existing National Institute for Health and Care Excellence (NICE) appraisal. For this appraisal, estimates of cost-effectiveness were sensitive to the choice of extrapolating model for overall survival (OS), and a key critique of the original extrapolations was that they fell below those of the age-matched general population. The second objective is to perform a simulation study, informed by the appraisal, to compare the performance of relative survival models.

## Methods

The code used for both the case-study and simulation study is available online (https://github.com/BenKearns/RelativeSurvival) and provides additional information.

### Case Study: Squamous Non–small-cell Lung Cancer

The existing HTA was a submission to NICE as part of their TA program.^18^ A NICE committee considers both the company submission and the independent evidence review group (ERG) critique of this as part of their decision-making process. The NICE committee provides recommendations on whether the technology is judged to be cost-effective and hence whether the technology should be recommended for routine use. For this appraisal, the population of interest was people with previously treated locally advanced or metastatic (stage IIIB or IV) squamous non–small-cell lung cancer. The intervention was nivolumab, and the sole comparator in the company’s submission was docetaxel. The main evidence source was the phase III trial CheckMate-017 (NCT01642004), which compared nivolumab (*n* = 135) against docetaxel (*n* = 137) for the population of interest (whose previous treatment was with platinum combination chemotherapy).^19^ Patient follow-up was between 11 and 24 mo. At the end of follow-up, there had been 86 (63.7%) and 113 (82.5%) deaths in the nivolumab and docetaxel arms, respectively. The primary outcome measure was OS. Evidence on effectiveness came solely from this trial, and there was no treatment switching in the data used in the company’s original submission.

For both OS and progression-free survival (PFS), the company based their approach to extrapolation on the guidance in NICE TSD 14.^20^ The assumption of proportional hazards was checked both visually and via significance tests. The company considered both standard survival models and Royston-Parmar models (RPMs),^21^ with up to 2 internal knots modeled on the hazard, normal, and odds scales (corresponding to extensions of the Weibull, lognormal, and log-logistic models, respectively) and Akaike’s information criteria (AIC) for goodness of fit. For OS, the assumption of proportional hazards appeared to hold, with a log-logistic model used for docetaxel. The treatment effect for nivolumab was modeled as a fixed hazard ratio of 0.59. For PFS, the proportional hazards assumption was judged to be violated. Hence, the company modeled both treatments using an RPM with 2 internal knots on the hazard scale. The probabilistic base-case incremental cost-effectiveness ratio (ICER) arising from this approach was £86,000 (all ICERs discussed in this article are given to the nearest £500 and are per quality-adjusted life-year gained), with a survival gain of 1.31 y for nivolumab.^18^ This value was robust to alternative approaches to extrapolation for PFS but not for OS. For example, when varying the hazard ratio across its plausible range, the ICER varied from £55,000 to £169,000.

The independent ERG were critical of the company’s OS extrapolations, in particular the fact that the extrapolated hazard eventually fell below that of the age-matched general population was deemed to be “wholly implausible, and inconsistent with any clinical evidence of treating metastatic disease.”^22^ The ERG contended that the extrapolated hazard for OS was likely to increase over time due to aging. Despite this, they extrapolated a constant hazard over time (using an exponential model). This was fit from 40 wk (9.2 mo) of follow-up (a temporal subset of the data), with the ERG suggesting that this cutoff was supported by the data. The ERG’s approach to OS extrapolation increased the company’s base-case ICER from £86,000 to £132,000, whereas the estimated lifetime survival gain more than halved, from 1.31 to 0.64 y. In response, the company amended their extrapolation approach to cap the extrapolated hazard rate so that it never fell below that of the corresponding general population. The company’s revised base-case ICER was £92,000, with a survival benefit of 1.16 y.^23^ However, the ERG remained critical of the company’s revised approach as not reflecting an anticipated long-term increase in hazards due to the effect of aging.^24^

Hence, the approach to extrapolating OS was identified as both a key area of uncertainty and a key driver of estimates of cost-effectiveness. The company fit survival models to all the available data and extrapolated a decreasing trend in the hazard. In contrast, the ERG fit a survival model to a subset of the available data and extrapolated a constant value (no trend), while also criticizing the company’s original extrapolations for eventually falling below that of the age- and sex-matched general population. The company in turn criticized the ERG’s approach as ignoring the trend in the hazard observed in the trial and lacking robustness by not using all the available data.

### Case Study: Reanalysis of the Clinical Effectiveness Data

Data on OS were digitized from the pivotal trial publication^19^ using the Engauge digitizer.^25^ These digitized data were used to replicate the original individual patient data using the algorithm of Guyot and colleagues.^26,27^ For consistency with the original company submission, initially both current practice and RPMs are considered for the docetaxel arm (providing the baseline hazard function), with DSMs introduced later. A fixed hazard ratio is used for the nivolumab treatment effect.

Within-sample goodness of fit is measured using AIC (there were no substantial differences when using Bayesian information criteria). Another measure, the inverse evidence ratio (IER) is also used to facilitate model comparisons. The IER is a measure of how plausible a model is, relative to the `best’ model (which has the minimum information criteria). Let $IC_{m}$ be the information criteria value (such as AIC) for model m , with minimum value $IC^{*}$ . The IER for model m is then $\exp(-0.5\;*[IC_{m}-IC^{*}])$ and will be 100% for the best fitting model, whereas values for poorly fitting models will be close to zero.^28^ Hence, the IER provides an interpretable scale for comparing model fit. Values are shown in Supplementary Tables S1 and S2 and demonstrate that the log-logistic model is the best fitting for both the standard models and the RPMs. Estimates of the hazard function from the second-best fitting RPM (4 internal knots, odds scale, results not shown) were visually very similar to the log-logistic model for both the within-sample and extrapolated periods.

**Table 1:** Cost-Effectiveness Estimates from Different Extrapolation Approaches.

	Absolute Value		Incremental Value		ICER (per QALY)
	QALYs	Cost	QALYs	Cost	
Replicated submission (no cap)					
Nivolumab	1.29	£85,882	0.74	£65,470	£87,926
Docetaxel	0.55	£20,413			
Replicated submission (with cap)					
Nivolumab	0.95	£72,943	0.39	£54,412	£139,958
Docetaxel	0.56	£18,530			
Replicated ERG approach					
Nivolumab	0.66	£56,985	0.33	£40,799	£124,807
Docetaxel	0.34	£16,186			
Dynamic survival models					
Local trend					
Nivolumab	1.06	£75,060	0.50	£56,699	£113,170
Docetaxel	0.56	£18,361			
Damped trend					
Nivolumab	0.87	£67,328	0.35	£49,600	£141,236
Docetaxel	0.52	£17,728			
Dynamic relative survival models					
Local level					
Nivolumab	0.88	£67,880	0.36	£50,229	£139,657
Docetaxel	0.52	£17,651			
Local trend					
Nivolumab	0.99	£72,990	0.45	£54,847	£122,328
Docetaxel	0.54	£18,143			
Damped trend					
Nivolumab	0.86	£66,899	0.34	£49,196	£142,825
Docetaxel	0.52	£17,702			

ERG, evidence review group; ICER, incremental cost-effectiveness ratio = incremental costs/incremental QALYs; QALY, quality-adjusted life-year.

Two DSMs are evaluated: a local trend and a damped trend model (see the supplementary material for model specification). Both may be viewed as modeling the log-hazard as a linear function of log-time. They differ with regard to the behavior of their extrapolations; a local trend model extrapolates the trend in the log-hazard indefinitely, whereas for the damped trend model, the extrapolated trend decreases as the extrapolation time horizon increases. Three DRSMs were evaluated: local trend, damped trend, and local-level implementations. These models assume that the observed trend continued until the excess hazard became zero, the observed trend continued in the short term (with long-term constant values of the excess hazard), and that the excess hazard was constant, respectively (see the supplementary material for full descriptions). As DRSMs formally incorporate external evidence on general population mortality, they are anticipated to provide more plausible extrapolations than DSMs for this case study.

To perform cost-effectiveness analyses, the company’s three-state partitioned survival analysis economic model was replicated in R, assuming a (lifetime) 20-y time horizon with a 1-wk time cycle. Utility data and resource use were primarily taken from CheckMate-017.^18^ The 2 alive health states of “stable” and “progressed” disease were assigned utilities of 0.750 and 0.592 (with standard deviations of 0.236 and 0.315), respectively. Everybody started in the stable health state. Results are based on a probabilistic sensitivity analysis with 2,000 iterations to account for nonlinearities in the model inputs. The model structure and inputs matched those reported in the original appraisal.^18^ Further details on the health economic model are provided in the supplementary material.

### Simulation Study

An additive hazards relative survival log-logistic model was used as the data-generating mechanism for the simulation study. To ensure that this mechanism was clinically plausible, it was obtained by fitting a log-logistic model to the case-study data (docetaxel arm), simulating from this model, and incorporating the (age-matched) general population hazard. For each individual, 3 times were simulated: a survival time from the log-logistic model, a survival time from the general population hazards (assuming a uniform distribution of deaths within a year), and a censoring time uniformly distributed between 5 and 6 y. This length of follow-up was chosen to ensure that there was sufficient data that included the turning point in the hazard function. The observed survival time was set to the minimum of the 3 sampled times (with event status similarly set). For this study, 200 simulations were performed, with each having a sample size of 300. Estimates of the “true” hazard function were based on the mean of 10 million simulations. Five models were considered: a log-logistic relative survival model, DRSMs with either a local or damped trend, and 2 flexible relative survival models. These use cubic splines to model the excess hazard and vary with how the model is specified. One uses the specification introduced by Nelson and colleagues (hereafter “Nelson relative survival” [NRS]); the other may also be written as a flexible mixture cure model. For both models, further details are provided by Jakobsen and colleagues.^15^ As the data-generating mechanism used a log-logistic model, the 2 DRSMs and the 2 flexible relative survival models were incorrectly specified, whereas the log-logistic relative survival model was correctly specified.

The estimand was the mean of the natural logarithm of the time-varying hazard function. The primary performance measure used was the mean (of the) squared error (MSE), with bias as a secondary performance measure. For MSE, smaller values indicate better model performance; for bias, this is indicated by values closer to zero. To avoid results being unduly influenced by implausibly large extrapolations, hazard estimates were capped to not exceed 1. Bias may be viewed as estimating how close to the truth estimates are on average, whereas MSE measures both bias and variability in estimates. Further details on the performance measures are available in the supplementary material.

## Results

### Case Study

**Figure 1:**
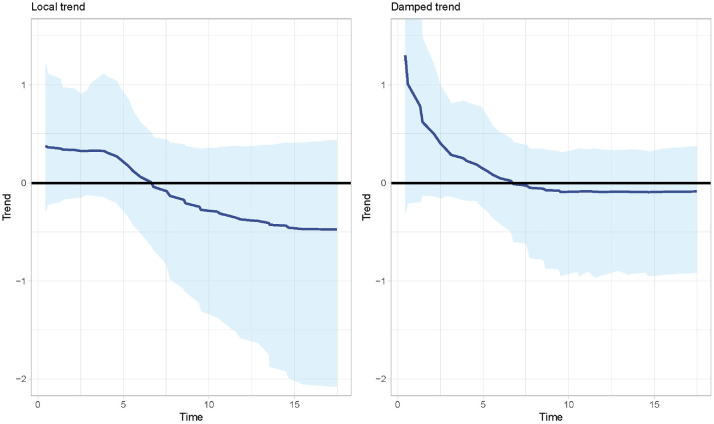
Estimates of the trend in the hazard function from 2 dynamic survival models. Solid blue line: point estimates, with 95% confidence intervals in pale blue. Black line: no trend.

Estimates of the trend in the hazard function over time, along with the uncertainty in these estimates are shown in Figure 1 for the 2 DSMs. This is of particular importance as there was disagreement over the assumed trend at the end of follow-up, with the company modeling a decreasing trend and the ERG modeling no trend. The trend estimate from both DSMs is initially positive followed by a decrease. For both models, the trend becomes negative at about half a year. For the local trend model, the trend estimates continue to decrease, albeit with a large degree of uncertainty. For the damped trend model, the trend is almost zero after half a year, suggesting that after this time, the assumption of a constant hazard may be appropriate. Figure 1 suggests that models that assume monotonicity (such as the Weibull and Gompertz) are inappropriate. In contrast, the use of a log-logistic or lognormal model may be acceptable, as the hazards from these can increase then decrease. Furthermore, the confidence intervals from both models include zero at all time points, indicating that a constant hazard model cannot be ruled out.

**Figure 2:**
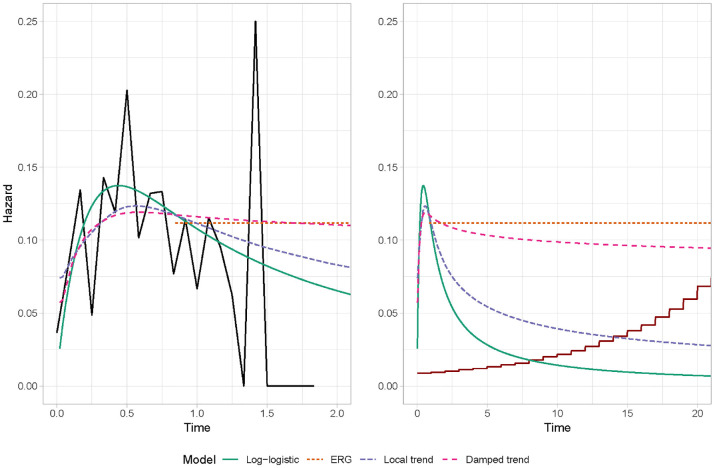
Hazard estimates without external data. Left: within-sample, right: extrapolations. Black line: observed hazard. Red line: general population hazard.

A visual comparison of the fit from the 2 DSMs along with the original company approach (log-logistic) and ERG approach (hybrid exponential) is provided in Figure 2. The observed hazard is generally unimodal, albeit with large variability due to small patient numbers toward the end of follow-up. For extrapolations, estimates of the annual hazard of all-cause mortality for the age-matched general population are also included based on 2016 UK data from the Human Mortality Database,^29^ assuming a starting age of 63 y (the median age of participants in CheckMate 017). For the first year of follow-up, estimates of the hazard function from the log-logistic and 2 dynamic models are visually similar, albeit the peak in the hazard is more pronounced for the log-logistic. At 1 y of follow-up, there are only 30 people still at risk (22% of the starting sample); this small sample size may be driving the differences in model estimates after 1 y. These differences continue into the extrapolated phase, with the largest decreases in the hazard function observed for the log-logistic model. In contrast, the damped trend model extrapolates almost constant hazards; in the short term, these estimates are very similar to those from the ERG approach, but they become increasingly smaller than the ERG extrapolations as the time horizon increases. Extrapolations from the local trend model lie between the log-logistic and damped trend models, eventually falling below age-matched general population estimates at approximately 15 y, hence potentially lacking face validity.

**Figure 3:**
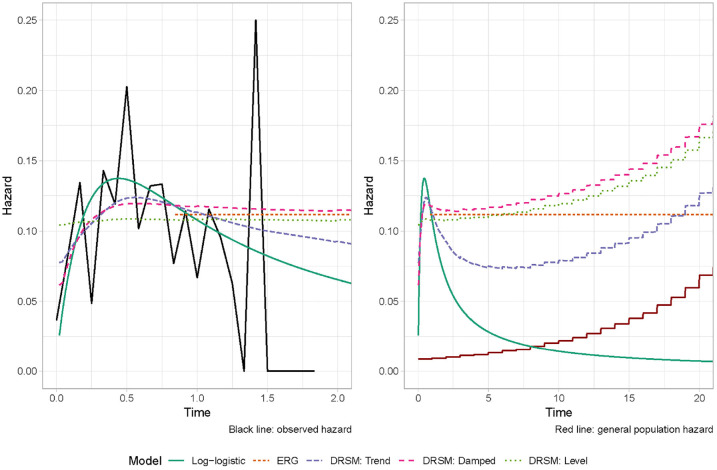
Hazard estimates with external data. Left: within-sample, right: extrapolations. Black line: observed hazard. Red line: general population hazard.

Estimates from DRSMs are shown in Figure 3, along with the log-logistic model and ERG approach for comparison. Visually, the local-level DRSM provides similar within-sample estimates to an exponential model and does not fit the data as well as the other models. Extrapolations from the local level and damped trend DRSMs are very similar to each other, illustrating that (as with the damped trend DSM) there is a pronounced dampening of the trend before the end of follow-up. After 20 y, hazards from all the DRSMs are greater than the general population estimates, implying that there is a nonnegligible extrapolated excess hazard. After about 10 y, the local trend DRSM extrapolates an increasing hazard, suggesting that after this point, the influence of aging on the hazard function outweighs the extrapolated decrease in the excess hazard.

Supplementary Table S4 compares the replication with the original company submission (using their approach to extrapolation) with the replicated model. Given that the individual patient-level data were re-created, there is in general close agreement, albeit with some underestimation of absolute costs. This is expected, as it was not possible to include a drug acquisition cost for the progressed disease health state. Cost-effectiveness results from the dynamic models are provided in Table 1. For comparison, 3 replicated analyses are also shown:

- The company’s original submission (extrapolation with a log-logistic model)
- Above, with extrapolated hazards capped by general population hazards
- The ERG’s hybrid approach (use Kaplan-Meier estimates up to 40 wks, extrapolations based on an exponential fit to the remaining data)

As shown in Table 1 and Figures 2 and 3, extrapolations can differ between the 5 dynamic models, which affects the cost-effectiveness results. The smallest ICER occurs for the local trend DSM (£113,000). The largest ICERs arise from both damped trend dynamic models and the local-level DRSM (£140,000 to £143,000). These 3 models all extrapolate a near-constant hazard. Variation in ICERs across the 3 DRSMs (£122,500 to £143,000) was slightly greater than variation between the ERG approach (£125,000) and the company submission with a cap (£140,000). Advantages of the DRSMs are first that model choice may be guided by clinical input into the likely behavior of the long-term excess hazard and, second, that external evidence is formally included as part of the model fitting procedure, instead of via a post hoc adjustment. Collectively, this allows for a stronger emphasis on understanding the likely behavior of the long-term excess hazard function and the plausibility of different assumptions about this long-term behaviour. As noted, an advantage of dynamic models over hybrid models is the avoidance of the subjective choice of which data to use for the extrapolating model. Estimates of cost-effectiveness can be sensitive to this choice, as illustrated in Supplementary Figure S1. Dynamic models also use all the data; with the ERG approach, only a third of the original sample (45 people) contribute to extrapolations.

### Simulation Study

**Figure 4:**
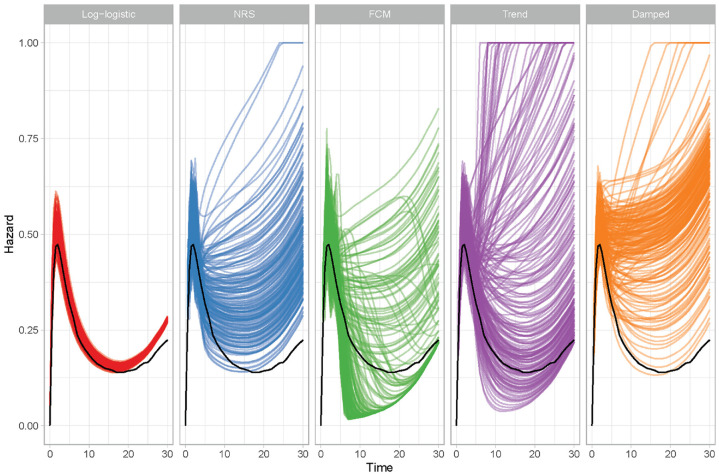
Relative survival model estimates of the hazard function and true values (black lines). FCM, flexible cure model; NRS, Nelson relative survival.

A graph of the true hazard function and the simulations from this is provided in the supplementary material (Supplementary Figure S2), whereas a visual comparison of model estimates with the truth is given in Figure 4. The correctly specified log-logistic relative survival model has the smallest variation in extrapolations, but there is a persistent overestimation that becomes more pronounced as the extrapolation time increases. Of the 2 flexible models (NRS and flexible cure model [FCM]), the NRS tends to overestimate the true hazard function, while the FCM underestimates it. Of the 2 dynamic models, the damped trend model has less variability in extrapolations, because of the dampening of the trend. However, this dampening means that, often, the decrease in the excess hazard is not captured, leading to overestimation. All of the flexible and dynamic relative survival models produce highly variable extrapolations, especially when compared with the log-logistic relative survival model.

Summary MSE and bias values are provided in Table 2, with plots of these statistics over time provided in Supplementary Figure S3. Consistent with Figure 4, the log-logistic model has the smallest variance, smallest bias, and lowest MSE values of all the relative survival models considered. Of the incorrectly specified models, MSE values were smallest for the NRS and trend DRSM (values of 0.086 and 0.089, respectively) and largest for the FCM (0.202). The trend DRSM had the smallest bias (0.127); however, there was a lot of uncertainty in the bias estimates, with each model’s confidence interval including the bias point estimate for every other model (including the log-logistic).

**Table 2:** Mean Squared Error and Bias Values, Averaged over Time.

Relative Survival Model	Mean Squared Error: Mean (95% CI)	Bias: Mean (95% CI)
Log-logistic	0.022 (0.020, 0.023)	0.106 (0.017, 0.195)
Nelson relative survival	0.086 (0.084, 0.089)	0.166 (0.031, 0.300)
Flexible cure model	0.202 (0.194, 0.211)	0.174 (0.039, 0.309)
Trend dynamic survival	0.089 (0.086, 0.092)	0.127 (0.059, 0.194)
Damped dynamic survival	0.122 (0.121, 0.124)	0.176 (0.103, 0.249)

## Discussion

A motivating case study introduced DRSMs and demonstrated the usefulness of relative survival models when extrapolating survival data. This case study informed a simulation study, which was used to compare different relative survival models. Flexible and dynamic relative survival models did not perform as well as the true model but are a potentially useful approach when the true survival model is unknown. The case study illustrated several benefits of dynamic models. This includes combining flexible fit to the observed data with explicit modeling of the long-term trend, incorporating external data to inform extrapolations, and encoding clinical views on long-term survival via model specification.

The clinical plausibility of extrapolations is very important. Additive relative survival models ensure that the extrapolated hazard function does not fall below that of the general population. This is not the only measure of extrapolation plausibility, but it is an important one that should be considered. Different model specifications are possible for DRSMs, reflecting different assumptions about the long-term behavior of the excess hazard. This flexibility in model specification and the direct interpretation of the extrapolations is a significant advantage of DRSMs when compared with other survival models and allows for the natural inclusion of clinical opinion about both the natural history of the disease and the likely mechanism of action of treatments. Basing model choice on clinical input into the natural history of the disease is of particular benefit, as good within-sample goodness of fit is not a predictor of good extrapolation performance.^30^ A further advantage of DRSMs is that it is straightforward to extend these to incorporate time-varying treatment effects that act on the disease-specific (excess) hazard function (see the supplementary material for specification). As the focus of the article was on different relative survival models, this extension was not pursued further, but it is noted that modeling the treatment effect as applying to the overall hazard can lead to biased results, as it includes the unrealistic assumption that treatment will reduce mortality that is unrelated to the disease.^31^ In addition, an alternative to modeling a treatment effect is to fit separate models to each arm; this will create an implicit treatment effect, and the plausibility of any such effects should be explicitly considered.

Re-created patient-level data were used in the case study. The re-created company submission showed close agreement with the original submission, demonstrating the usefulness of using re-created data. One limitation was that it was not possible to explore the effects of covariates on survival. In particular, when estimating relative survival, it has been demonstrated that including age can lead to increased accuracy.^32^

The case-study results from the DRSMs suggest that the ICER arising from the company’s original approach (£88,000) is likely to be too low; depending on the long-term prognosis of patients, the ICER is likely to be between £122,000 and £143,000. This range of ICERs is above the acceptable threshold for end-of life treatment, which is typically assumed to be £50,000. Following their original submission, the company offered a discount to the cost of their treatment to lower the ICER (and so improve the possibility of a positive recommendation). The magnitude of discount required to make the treatment cost-effective will be strongly affected by the extrapolation approach used. Of the approaches evaluated here, it is not possible to definitively state which would be the preferred base-case analysis, but the use of a dynamic model that incorporates external evidence appears to be the most useful. Future research could identify the situations in which the different DRSM specifications (including the modeling of the treatment effect) are the most appropriate. Relative survival models that do not bound the overall hazard by the general population hazard are also possible.^16^ There is uncertainty whether long-term extrapolated hazards should be bounded by the general population hazards (that is, if long-term survivors have a better prognosis than the general population does); long-term follow-up from trials would be able to provide insight into this.

For the simulation study, the correctly specified log-logistic relative survival model provided the best extrapolations of the models considered. Alternative standard parametric relative survival models were not considered, as these typically have strong parametric assumptions. For example, the Weibull model assumes that the excess hazard is monotonic, which is known to be inadequate for the simulation study. In practice, the suitability of a model with monotonic hazards may be unknown; similar work on model choice for cure models has shown that for standard parametric models, extrapolations can be sensitive to model misspecification.^11^ The alternative relative survival models considered in the simulation study have very weak structural assumptions, and so model misspecification is less of an issue. However, these models can provide highly variable extrapolations. Further research into reducing the variability of these extrapolations will be very useful. This could involve the use of other types of external evidence, such as registry data or previous trials for the disease of interest.^33^ Further research could also identify whether there are certain situations in which 1 or more of the models considered are of particular benefit. There were some limitations to this simulation study. Only a single data-generating mechanism was considered; future work could consider different designs such as model specification, censoring mechanism, sample size, and length of follow-up. The length of follow-up used here (5 to 6 y) was longer than is often seen in HTA, which suggests that in practice, relative survival models may perform even worse for extrapolation. The comparative performance, as evaluated here, should, however, remain similar.

In conclusion, survival data describe the occurrence of deaths over time and so form a natural time series. This motivates the use of dynamic models, which can exploit the temporal evolution of the hazard function when generating extrapolations. These models combine flexible within-sample estimates with parsimonious models for extrapolations that have meaningful clinical interpretations. These models, along with relative survival models that incorporate external evidence on general population mortality, have potential advantages over the survival models currently used in HTA. In the simulation study of this article, dynamic and flexible relative survival models had similar extrapolation performance. These models impose minimal structural assumptions and can provide good within-sample estimates. Further experience of these models is required to provide more specific guidance about the role of both dynamic models and relative survival models in HTA.

## Supplemental Material

sj-docx-1-mdm-10.1177_0272989X221107649 – Supplemental material for Dynamic and Flexible Survival Models for Extrapolation of Relative Survival: A Case Study and Simulation Study Click here for additional data file. Supplemental material, sj-docx-1-mdm-10.1177_0272989X221107649 for Dynamic and Flexible Survival Models for Extrapolation of Relative Survival: A Case Study and Simulation Study by Benjamin Kearns, Matt D. Stevenson, Kostas Triantafyllopoulos and Andrea Manca in Medical Decision Making
