# Supplementary material for: Dynamic and Flexible Survival Models for Extrapolation of Relative Survival: A Case Study and Simulation Study
Source: Med Decis Making. 2022 Jun 29;42(7):945–55. doi: 10.1177/0272989X221107649 (PMC9459356; doi:10.1177/0272989X221107649)

# Supplementary Appendix

## Dynamic relative survival models: technical details

A dynamic relative survival model (DRSM) extends a dynamic survival model to incorporate evidence on general population mortality. The specification of a dynamic survival model is:

$$Y_{t_{i}}\sim\text{Poisson}\left( \exp\left( \beta_{1,t_{i}} \right)\tau_{t_{i}} \right)$$

$$\beta_{1,t_{i}}=\beta_{1,t_{i-1}}+\beta_{2,t_{i-1}}\phi\omega_{t_{i}}$$

$$\beta_{2,t_{i}}=\beta_{2,t_{i-1}}\phi+\zeta_{t_{i}}$$

$$\zeta_{t_{i}}\sim N\left( 0,Z \right)$$

Where $Y_{t_{i}}$ is the number of deaths in the interval starting at time $t_{i}$ and ending at time $t_{i+1}$ and $\tau_{t_{i}}$ is the ‘at risk’ sample in this interval, for which it is assumed that censored events occur half-way through the interval whilst deaths occur at the end. The latent variables $\beta_{1,t_{i}}, \beta_{2,t_{i}}$ may be interrepted as the average log-hazard and trend (both at time $t_{i}$). The interval width is denoted by $\omega_{t_{i}}$, whilst $0\leq\phi\leq1$ is a dampening parameter; the lower the value of $\phi$ the more the extrapolated trend is reduced (dampened). Finally, $\zeta_{t_{i}}$ is the error term (innovation), assumed to be an independent and identically distributed series with variance $Z$.

To extend the above to a DRSM, let $\lambda_{t_{i}}^{P}, \lambda_{t_{i}}^{E}$ denote the general population hazard and the disease-specific (excess) hazard respectively (both at time $t_{i}$). The only change in model specification is for the number of deaths;

$$Y_{t_{i}}\sim\text{Poisson}\left( \left[ \exp\left( \beta_{1,t_{i}} \right)+\lambda_{t_{i}}^{P} \right]\tau_{t_{i}} \right)$$

For this, $\exp\left( \beta_{1,t_{i}} \right)=\lambda_{t_{i}}^{E}$, so may now be interpreted as modelling the log-excess hazard. The dynamic part of the model now relates to the evolution of the disease-specific hazard over-time. This is of particular use, as it is expected that the natural history of a disease will be driven by changes in the disease-specific hazard, which in turn will be affected by the treatment received. Choice of the specific DRSM to use may then be driven by clinical input into the likely long-term behaviour of the disease-specific hazard beyond the end of the available data. The impact of alternative assumptions may be tested by assessing alternative DRSMs in scenario analyses. There are three options of particular interest:

- The excess hazard decreases to zero. This would be modelled with a local trend model (setting $\phi=1$). It assumes that individuals who are still alive after a certain time-point may be viewed as `cured' from their disease, as their subsequent survival matches that of the general population. The time until this `cure' occurs is determined by the observed data.
- There is a constant excess hazard. This is represented by a local level model (setting $\beta_{2,t_{i}}=0=\phi$ and instead modelling variation in the $\beta_{1,t_{i}}$). This model is appropriate if it is assumed that individuals will always be at an increased risk of death compared to the general population, and that this risk is unlikely to vary over time.
- The excess hazard decreases in the short-term, to a constant value. A damped trend model would be suitable in this instance. This model assumes that individuals will never be cured from their disease but that the impact of the disease on their chances of dying decrease over time.

Note that the interpretation of the local trend and damped trend models above assumes that the extrapolated trend is decreasing. If it is increasing then these two models assume that the observed increase in the excess hazard will either persist indefinitely or increase over time to a constant value, respectively.

The DRSM specified here assumes that the overall observed hazard is an additive composition of the general population and disease-specific hazards. Alternative assumptions are possible, in particular if assuming a multiplicative composition, then for values of $\lambda_{t_{i}}^{E}$, the overall hazard would be less than the general population hazard. The DRSMs presented here are designed to constrain the overall hazard to never fall below the general population hazard, so only the additive specification is considered.

A DRSM may be extended to incorporate a time-varying treatment effect. The model is then:

$$Y_{t_{i}}^{(j)}\sim\text{Poisson}\left( \left[ \exp\left( \beta_{1,t_{i}}+j\delta_{1,t_{i}} \right)+\lambda_{t_{i}}^{P} \right]\tau_{t_{i}} \right)$$

$$\beta_{1,t_{i}}=\beta_{1,t_{i-1}}+\beta_{2,t_{i-1}}\phi\omega_{t_{i}}$$

$$\beta_{2,t_{i}}=\beta_{2,t_{i-1}}\phi+\zeta_{t_{i}}^{\beta_{2}}$$

$$\delta_{1,t_{i}}=\delta_{1,t_{i-1}}+\delta_{2,t_{i-1}}\phi\omega_{t_{i}}$$

$$\delta_{2,t_{i}}=\delta_{2,t_{i-1}}\phi+\zeta_{t_{i}}^{\delta_{2}}$$

$$\zeta_{t_{i}}^{\beta_{2}}\sim N\left( 0,Z \right)$$

$$\zeta_{t_{i}}^{\delta_{2}}\sim N\left( 0,Z \right)$$

Where $j$ is the group indicator (here = 0 for those receiving docetaxel and 1 for those receiving nivolumab), $Y^{(j)}$ are the observed deaths for group $j$, $\delta_{1,t_{i}}$ and $\delta_{2,t_{i}}$are the level and trend for the treatment effect, and the trend has innovation variances $\zeta_{t_{i}}^{\delta_{2}}$ (the innovation variance for $\beta_{2}$ has been similarly amended). As with the excess hazard, different models may be used to reflect different assumptions about the behaviour of the treatment effect during the extrapolated period.

## Simulation study: additional details

The estimand was the mean natural logarithm of the time-varying hazard function $\lambda_{t_{i}}$. The natural logarithm was used. This maps $\lambda_{t_{i}}$ to the range $\left( -\infty,\infty\right)$ and it may be assumed to be approximately Normally distributed. Hence, both positive and negative deviations would be equally likely.

When the estimand is the mean, and positive and negative deviations from the mean are penalised equally, then the squared error is a consistent loss function, or performance measure (25). Here consistency means that the performance measure is minimised when model estimates equal the estimand. The primary performance measure used was the mean (of the) squared error (MSE), with bias as a secondary performance measure. In addition to being a consistent loss function, the MSE has the benefit that it may be interpreted as penalising for both bias (how close are the model estimates to the truth) and variance (how much do estimates vary across simulations). Use of bias as a secondary measure provides insight into how the two components of bias and variance contribute to the MSE. The MSE and bias are defined as (16):

$$\text{MSE}_{i}=\frac{1}{n_{\text{sim}}}\sum_{j=1}^{n_{\text{sim}}} \left( \hat{\theta_{j,i}}-\theta_{i} \right)^{2}$$

$$\text{Bias}_{i}=\frac{1}{n_{\text{sim}}}\sum_{j=1}^{n_{\text{sim}}} \left( \hat{\theta_{j,i}}-\theta_{i} \right)$$

Where $n_{\text{sim}}$ is the number of simulations, $\theta_{i}$ is the estimand, and $\hat{\theta}_{j,i}$ is the corresponding model-based estimate (subscripts i, j denote time and simulation). For MSE lower values indicate better model performance, for bias values closer to zero indicate better model performance. As the hazard function is a time-varying estimand the performance measures are also time-varying. Summary (mean) values of the MSE and bias were calculated separately for the out-of-sample (extrapolations) and within-sample time periods. These summary measures use a novel method to calculate; the values of interest are:

$$f(x)=\frac{1}{T}\sum_{i=1}^{T} \mu_{i}$$

Where $\mu_{i}$ is the MSE or bias (with corresponding standard deviation of $\sigma_{i}$). Whilst $E\left[ f(x) \right]$ is a weighted mean, there is no standard expression to calculate $\text{Var}\left[ f(x) \right]$. Here a simulation-based approach is used. It is assumed that the MSE $\sim Gamma\left( \frac{\mu_{i}^{2}}{\sigma_{i}^{2}},\frac{\sigma_{i}^{2}}{\mu_{i}} \right)$ and the bias $\sim N\left( \mu_{i},\sigma_{i}^{2} \right)$. MSE and bias values were sampled 1,000 times from these distributions and used to generate percentile-based confidence intervals.

## Goodness of fit statistics

**Table S1: Goodness of fit statistics for the standard survival models.**

| **Model** | **Log-logistic** | **Lognormal** | **Generalized gamma** | **Gamma** | **Weibull** | **Exponential** | **Gompertz** |
| --- | --- | --- | --- | --- | --- | --- | --- |
| AIC | 721.3 | 723 | 723.6 | 724.9 | 726.9 | 730.8 | 732 |
| IER | 100% | 42% | 31% | 17% | 6% | 1% | 0% |

**Table S2: Goodness of fit statistics for the Royston-Parmar models.**

| **Interior knots** | **AIC: Hazard** | **AIC: Normal** | **AIC: Odds** | **IER: Hazard** | **IER: Normal** | **IER: Odds** |
| --- | --- | --- | --- | --- | --- | --- |
| None | 726.9 | 723.0 | 721.3 | 6% | 42% | 100% |
| One | 724.5 | 722.8 | 722.9 | 19% | 45% | 44% |
| Two | 724.4 | 724.0 | 724.8 | 21% | 25% | 17% |
| Three | 724.1 | 722.6 | 723.1 | 24% | 50% | 39% |
| Four | 722.4 | 722.7 | 722.4 | 55% | 48% | 57% |
| Five | 725.8 | 725.9 | 725.7 | 11% | 10% | 11% |

## Health economic model

The model perspective is that of the NHS and personal social services. Discounting of costs and benefits occurred at 3.5% per year. The year of costs is the same as that of the original appraisal; 2015 (this allows for a more direct comparison with the original cost-effectiveness results). The median age of participants in the pivotal CheckMate 017 trial was 63, which was taken to be the starting age in the model.

Progression-free survival was modelled using a Royston-Parmar model (on the hazard scale) with two internal knots. Hazard estimates from all models were converted to probabilities for use in the health economic model, and probabilities over time was constrained so that the probabilities of moving out of the progression-free health state (via either disease progression or death) were never smaller than the probability of death (at the corresponding time point).

An overview of the costs included are provided in Table S3. Nivolumab is dosed at 3 mg/kg and docetaxel at 75 mg/m^2^. Evidence on mean patient weight and body surface area were informed by CheckMate 017, giving values of 73kg and 1.82m^2^. Treatment was assumed to be until disease progression or death. Implementation of the probabilistic sensitivity analysis was based on the company approach of using a Gamma distribution, assuming that the standard error was 10% of the mean. For utilities, a beta distribution was used. In this replication, drug acquisition costs are only applied for the stable disease health state because evidence for progressed disease were submitted as confidential-in-confidence and hence were not publicly available.

**Table S3: Costs included in the health economic model.**

| **Description** | **Mean value (£)** |
| --- | --- |
| **Stable disease costs** | |
| Disease management (per 4 weeks) | 313.55 |
| Drug acquisition: nivolumab (per 2 weeks) | 2,634 |
| Drug acquisition: docetaxel (per 3 weeks) | 900 |
| Administration: nivolumab | 269.92 |
| Administration: docetaxel | 167.34 |
| Monitoring (per 4 weeks) | 151.89 |
| **Progressed disease** | |
| Disease management (per 4 weeks) | 766.62 |
| Drug acquisition cost | *Not reported* |
| **Transition to death** | |
| End of life care | 3,628.70 |

Data on the frequency of adverse events were also marked as commercial-in-confidence. A breakdown of results from the company’s submission states that, of the average discounted costs per patient, adverse events contributed £228 and £1,304 for nivolumab and docetaxel, respectively. As such, the average results from the replicated model were adjusted by these amounts to reflect the impact of adverse events. As with costs, a post-hoc adjustment was made to model outputs from the replicated model to reflect the impact of adverse events. These were to decrease the average QALYs by 0.01 and 0.05 for nivolumab and docetaxel, respectively.

Details on the distributions used for utility values in the company’s probabilistic analyses were not provided. Hence, the available data (mean and standard deviation) were used to derive parameters of the beta distribution using the method of moments.

## Additional results

**Table S4: Comparison of cost-effectiveness results: original submission and replication.**

|  | **Absolute Value** | | | **Incremental values** | | **ICER** |
| --- | --- | --- | --- | --- | --- | --- |
| **Deterministic results** | **Life Years** | **QALYs** | **Cost** | **QALYs** | **Cost** |  |
| *Original submission* |  |  |  |  |  |  |
| Nivolumab | 2.26 | 1.30 | £86,599 | 0.76 | £65,355 | £85,950 |
| Docetaxel | 0.95 | 0.54 | £21,243 |  |  |  |
| *De novo replication* |  |  |  |  |  |  |
| Nivolumab | 2.22 | 1.28 | £86,073 | 0.74 | £65,891 | £89,309 |
| Docetaxel | 0.95 | 0.54 | £20,182 |  |  |  |
| **Probabilistic results** | **Life Years** | **QALYs** | **Cost** | **QALYs** | **Cost** | **ICER** |
| *Original submission* |  |  |  |  |  |  |
| Nivolumab | NR | 1.35 | £91,677 | 0.77 | £68,938 | £89,343 |
| Docetaxel | NR | 0.58 | £22,739 |  |  |  |
| *De novo replication* |  |  |  |  |  |  |
| Nivolumab | - | 1.29 | £85,882 | 0.74 | £65,470 | £87,926 |
| Docetaxel | - | 0.55 | £20,413 |  |  |  |

ICER = incremental cost-effectiveness ratio = incremental costs / incremental QALYs. All values are discounted with the exception of life-years. NR: Not reported.

**Figure S1: Sensitivity of the ICER to different cut values (ERG extrapolation approach)**

[Footnote: ERG: Evidence review group. ICER: Incremental cost-effectiveness ratio.]


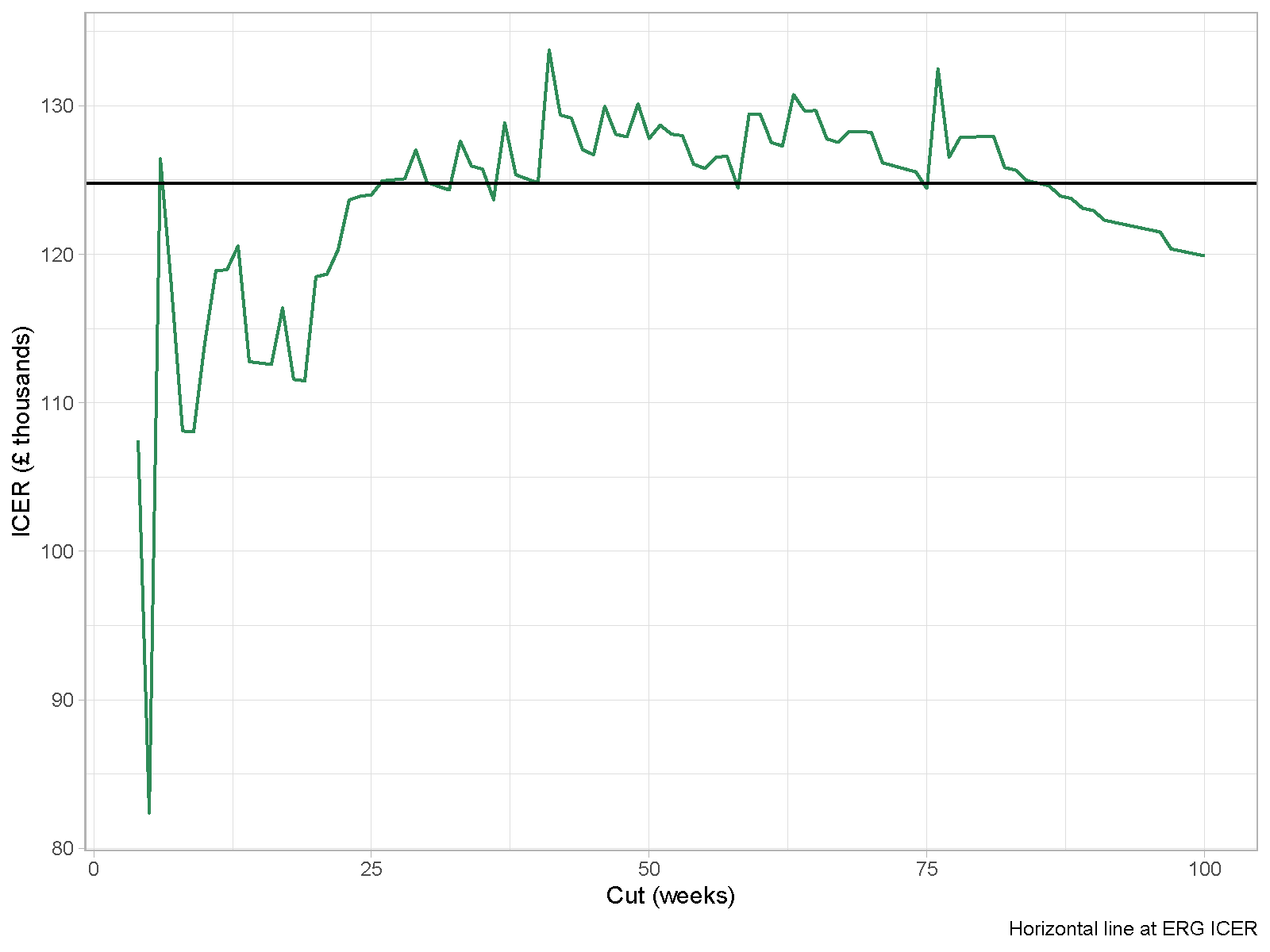


**Figure S2: True (black line) and simulated hazard functions (grey lines)**


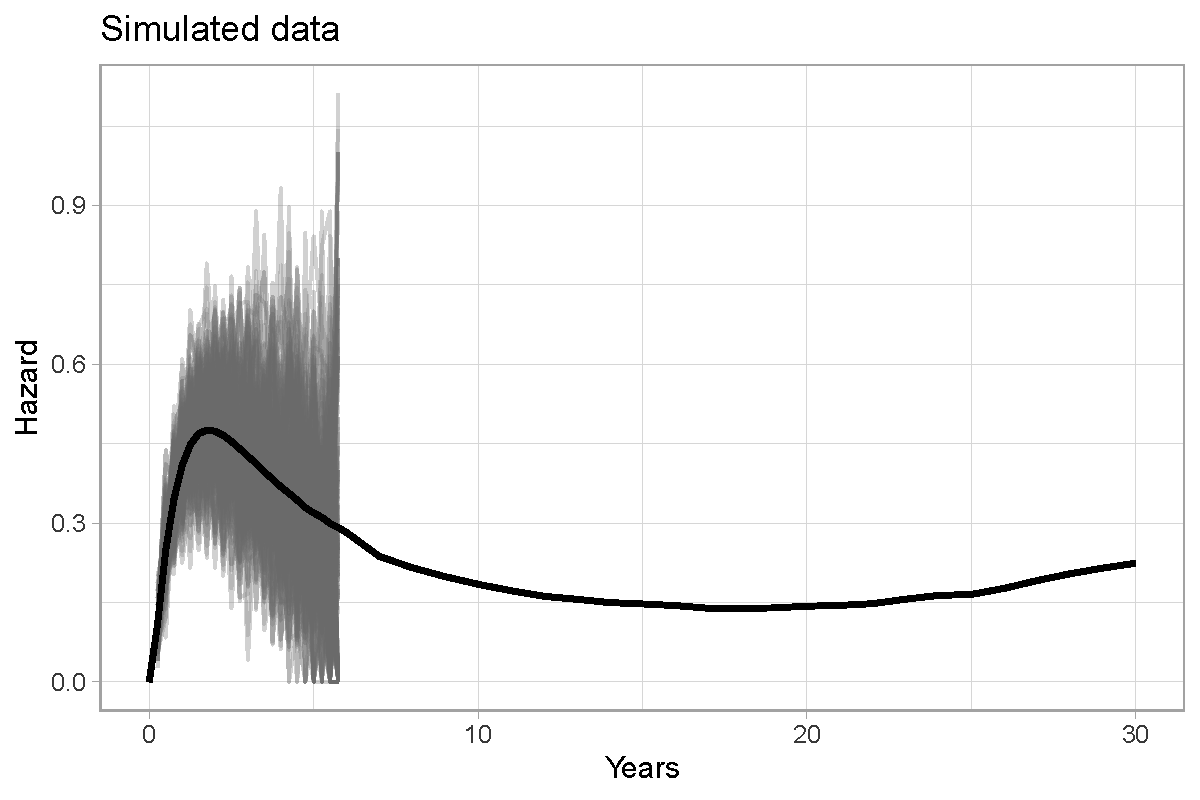


**Figure S3: Measures of fit (mean squared error and bias) over time, by model.**


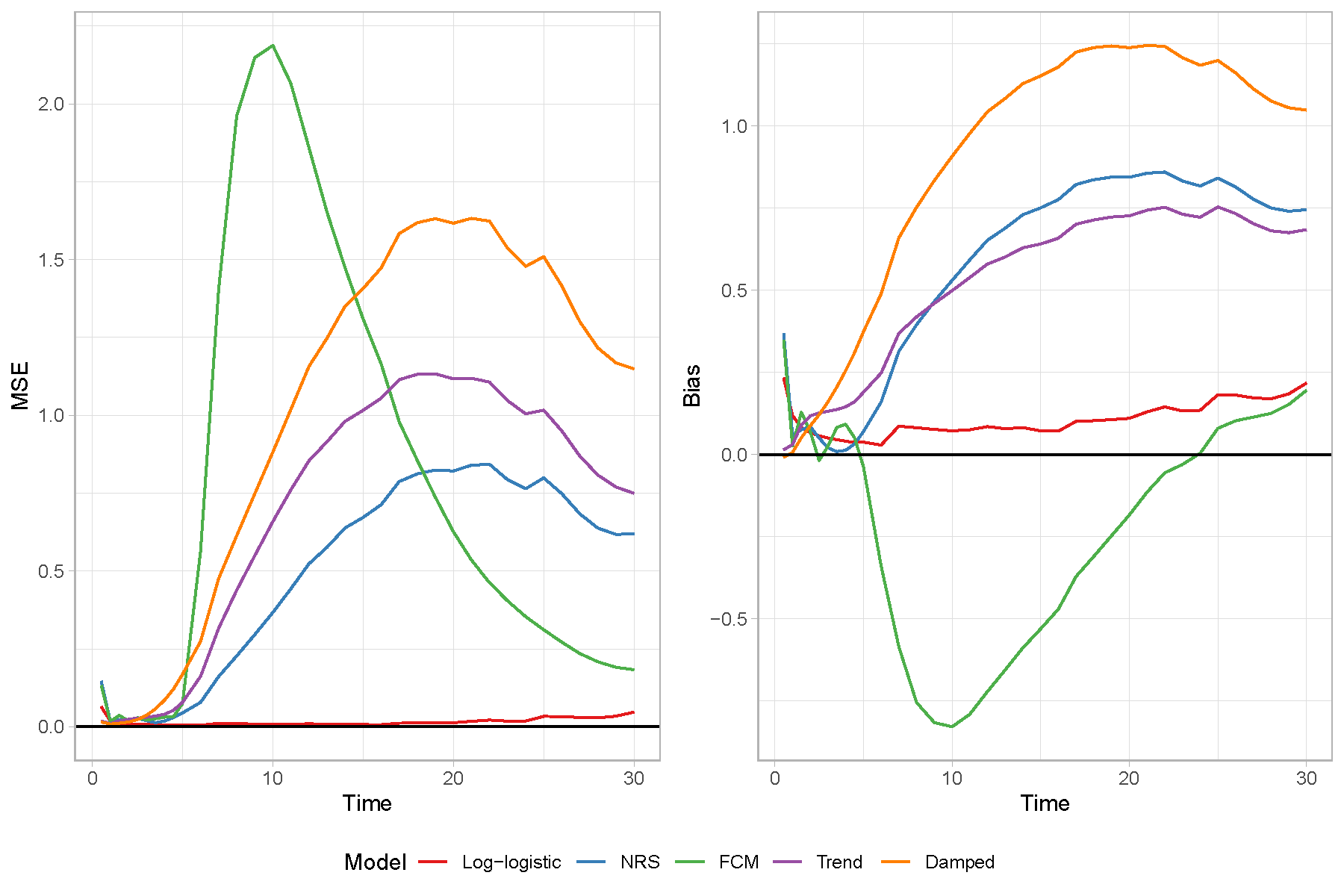

Supplement: sj-docx-1-mdm-10.1177_0272989X221107649 – Supplemental material for Dynamic and Flexible Survival Models for Extrapolation of Relative Survival: A Case Study and Simulation Study [file sj-docx-1-mdm-10.1177_0272989X221107649.docx]
